# Socioeconomic risk factors for labour induction in the United Kingdom

**DOI:** 10.1186/s12884-020-2840-3

**Published:** 2020-03-06

**Authors:** Sarah Carter, Amos Channon, Ann Berrington

**Affiliations:** 1 MRC Lifecourse Epidemiology Unit, University of Southampton, Southampton General Hospital, Southampton, SO16 6YD UK; 2 Social Statistics & Demography, Economic, Social & Political Sciences, University of Southampton, Southampton, SO17 1BJ UK

**Keywords:** Labour induction, Maternal health, Childbirth intervention, Health care

## Abstract

**Background:**

Labour induction is a childbirth intervention experienced by a growing number of women globally each year. While the maternal and socioeconomic indicators of labour induction are well documented in countries like the United States, considerably less research has been done into which women have a higher likelihood of labour induction in the United Kingdom. This paper explores the relationship between labour induction and maternal demographic, socioeconomic, and health indicators by parity in the United Kingdom.

**Method:**

Logistic regression analyses were conducted using the first sweep of the Millennium Cohort Study, including a wide range of socioeconomic factors such as maternal educational attainment, marital status, and electoral ward deprivation, in addition to maternal and infant health indicators.

**Results:**

In fully adjusted models, nulliparous and multiparous women with fewer educational qualifications and those living in disadvantaged places had a greater likelihood of labour induction than women with higher qualifications and women in advantaged electoral wards.

**Conclusions:**

This paper highlights which UK women are at higher risk of labour induction and how this risk varies by socioeconomic status, demonstrating that less advantaged women are more likely to experience labour induction. This evidence could help health care professionals identify which patients may be at higher risk of childbirth intervention.

## Background

The rate of labour induction has increased in the United Kingdom over the past 25 years, in all four countries in the UK. Labour induction has previously been associated with a greater risk of subsequent childbirth interventions [1–5], and, due to its link with operative births, it can be a costly intervention [6, 7]. Previous research has also found that women experiencing their first births are more likely to be induced [2]. It is therefore important to understand factors associated with labour induction, and how they might differ by parity between groups of women.

Past research on indicators of labour induction has tended to focus on medical risk factors such as the woman’s age, the presence of diabetes or hypertension, or the infant’s birth weight and gestational age [8–15]. This paper aims to determine whether socioeconomic factors such as maternal education, income, or local neighbourhood deprivation have independent associations with induction in the United Kingdom once medical factors are controlled.

Most of the research on the broader determinants of labour induction has been conducted in the United States. These studies indicate that women who are college-educated, white, and covered by commercial health insurance are the most likely to have their labours induced [8, 12]. However, the US does not have the universal health care that is established in the United Kingdom and therefore it is not obvious whether the US findings are generalizable. Literature from countries with universal health care suggests that women who undergo labour induction in those countries may differ from those who do in the United States. For example, Cammu et al. (2011) found that in Belgium, higher educational qualifications made women less likely to experience labour induction. This inverse relationship between maternal educational qualifications and childbirth intervention has also been reported in Norway by Tollånes et al. (2007) and in Canada by Stoll and Hall (2012).

Humphrey and Tucker (2009) is one of the few studies based in the UK that has examined social indicators of induction, utilizing data from one university hospital in Aberdeen, Scotland. They found that while medical risk factors (such as maternal age, parity and BMI) and a woman’s area of residence in Aberdeen were associated with labour induction, marital status and social class were not [16]. While Humphrey and Tucker (2009) explores the influence of residential area and adds to the literature citing BMI and parity as important maternal indicators of labour induction, other demographic indicators (such as maternal ethnicity) and markers of socioeconomic status (e.g. maternal income quintile and educational qualifications) are not examined.

Given that the relationship between labour induction and maternal socioeconomic indicators may differ in the UK, the aim of this paper is to develop an understanding of labour induction in the United Kingdom and to explore the role of maternal socioeconomic factors in labour induction risk, while accounting for medical risk factors. By so doing, this paper is able to provide insights into the influence of non-medical indicators of labour induction, which can help identify women who may need more support from their health care providers in making decisions about childbirth interventions.

## Methods

### The Millennium Cohort Study

The Millennium Cohort Study (MCS) is a longitudinal survey of over 19,000 cohort children born in 2000–2001 in the United Kingdom. This study provides one of the best opportunities to examine the predictors of labour induction, as it includes a wide range of information concerning the women’s socioeconomic and health backgrounds, their labour experiences, and birth outcomes. It also draws its clustered sampling frame from the whole of the UK and has been linked to contextual information on ward-level deprivation.

The sample consists of the natural mothers of surviving singleton births who were interviewed 9 months after the birth of the cohort member. As twin and triplet pregnancies are less likely to end in labour inductions, twin (246) and triplet (10) births were removed from the analysis, bringing the final sample size to 18,241. The analysis for the present paper split women into two groups, nulliparous (no previous births) and multiparous (one or more previous births), in an effort to illuminate any differences in the predictors of labour induction according to parity.

Ethical approval for the secondary data analysis presented here was granted by the University of Southampton in 2015.

### Outcome variable - induction

The dichotomous outcome variable was whether or not a woman had undergone any form of labour induction during the birth of the cohort member, with the survey question asking, “Was the labour induced or attempted to be induced? [Note: Induced labour = any attempt to start labour (including injections, pessaries, breaking the waters)]” [17].

### Explanatory variables

Demographic indicators included in the logistic regression models were age, ethnicity, and partnership status at birth. Maternal socioeconomic status was measured by highest level of educational qualification, occupation, household income quintile, and housing tenure.

Local area deprivation is coded as a composite variable created by the MCS, measuring the relative advantage or disadvantage of the area in which a respondent lived, and was derived using indices of multiple deprivation from the electoral ward level linked to the address at interview. Using deprivation data for the four UK countries under review obtained from the Office of National Statistics (ONS), the Welsh Assembly, the Northern Ireland Statistics and Research Agency (NISRA), and the Scottish government, the MCS organized households into nine categories: England – Advantaged; England – Disadvantaged; England – Ethnic; Wales – Advantaged; Wales – Disadvantaged; Scotland – Advantaged; Scotland – Disadvantaged; Northern Ireland – Advantaged; and Northern Ireland – Disadvantaged. In England, households were placed into the “High ethnic density” category if they were located in electoral wards with populations at least 30% identifying as “Black” or “Asian,” “Disadvantaged” if they were not categorized as having high ethnic density and were among the poorest 25% of wards based on the Child Poverty Index for England and Wales, and “Advantaged” if they did not fall into either of the above categories. In Wales, Scotland, and Northern Ireland, households were deemed “Disadvantaged” if they were among the poorest 25% of wards based on the Child Poverty Index for England and Wales, and “Advantaged” if they were not among the poorest 25%.

Medical risk factors included in the following analyses are maternal body mass index (BMI) before pregnancy, smoking behaviour in pregnancy, infant birth weight, and infant gestational age in days. Birth weight and number of gestational days were included in analyses in an attempt to control for babies who were small for their gestational age.

Women experiencing certain medical indications associated with labour induction, such as hypertension, diabetes, or restricted foetal growth, may be more likely to be induced than women without those complications. Therefore, health in pregnancy was controlled for in the following analyses. An extensive list of various pregnancy complications was collapsed into a variable with four categories:

1. No pregnancy complications
2. Pregnancy complications not usually associated with induction: threatened miscarriage, backache, vomiting, placental problems, accidents
3. Pregnancy complications associated with induction: raised blood pressure, eclampsia/preeclampsia, diabetes, gestational diabetes, too much or little fluid around the baby, suspected restricted foetal growth, liver/gall bladder problems, cholestasis, early rupture of the membranes
4. Other

### Model specification

Descriptive statistics were run to report the distribution of demographic, socioeconomic and health variables, and chi square analyses were performed to determine associations between labour induction and these explanatory variables. Multivariate logistic regression was used to calculate the log-odds of a woman having experienced labour induction during the birth of the cohort member. Two nested logistic regression models were fit following hypothesised relationships between the explanatory factors and induction:

1. Model 1: Maternal demographic and socioeconomic information
2. Model 2: Model 1 plus maternal and infant health indicators

Therefore, Model 2 controlled for all demographic, socioeconomic, and maternal and infant health variables noted above. These models allowed the strength of the relationship between labour induction and maternal socioeconomic indicators to be modelled both before and after the adjustment for health risk factors. Tests for multicollinearity were performed to ensure no variables were collinear in the fully adjusted Model 2 for nulliparous and multiparous women. Maternal BMI before pregnancy was the only covariate with missing data; sensitivity analyses determined that removing cases with missing maternal BMI data did not change the magnitude, direction, or significance of any of the relationships in any models run for either group of women. Trend was tested by re-running both Models 1 and 2 with the ordinal variables maternal age, maternal BMI, infant birth weight, and infant gestational age entered as linear variables. There was no change in the relationship between these variables and risk of labour induction. All analyses were conducted using STATA 14.

## Results

### Descriptive findings

The distribution of the variables used in the analysis is displayed in Table 1. A higher percentage of nulliparous women were induced (36.4%) than multiparous women (27.2%). While both groups of women had similar proportions of minority ethnic group membership, with the vast majority of respondents identifying as White, multiparous women tended to be older.

**Table 1:** Weighted Distribution of Variables Used in Regression Analysis of Labour Induction Among Nulliparous and Multiparous Women

		Nulliparous		Multiparous	
		%	Number	%	Number
Labour Induction	Not induced	63.6	4754	72.8	7817
	Induced	36.4	2721	27.2	2925
Age	19 years and under	18.0	1350	2.2	232
	20–24 years old	28.9	2163	20.1	2165
	25–29 years old	28.7	2148	30.6	3291
	30–34 years old	18.4	1378	31.6	3397
	35 years and older	6.0	451	15.5	1663
Ethnicity	White	86.0	6432	82.5	8855
	Indian	2.7	199	2.5	273
	Pakistani/Bangladeshi	5.2	386	8.1	868
	Black/Black British	2.9	216	4.2	449
	Other	3.3	243	2.7	290
Marital Status	Legally Married	50.7	3798	64.4	6925
	Cohabiting	27.9	2088	20.4	2195
	Unpartnered	21.4	1604	15.2	1631
Education	Higher/first degrees	19.0	1422	13.4	1436
	Diplomas in higher ed	9.4	703	7.6	819
	A/O Levels (GSCE A-C)	43.4	3244	42.4	4542
	Other (incl. GCSE D-G)	14.4	1073	13.2	1410
	None	13.8	1032	23.5	2514
Occupation	Managerial/professional	30.5	2283	22.9	2459
	Intermediate	18.8	1409	15.2	1636
	Self-employed	2.6	197	4.1	442
	Lower supervisor	5.2	390	5.6	599
	Semi-routine/Routine	33.0	2475	39.0	4197
	None	9.8	736	13.2	1418
Household Income Quintile	Lowest Quintile	23.0	1719	26.6	2848
	Second Quintile	17.4	1296	26.1	2797
	Third Quintile	18.5	1380	19.3	2064
	Fourth Quintile	19.1	1425	16.3	1744
	Highest Quintile	22.0	1645	11.8	1262
Housing Tenure	Own outright/mortgage	57.5	4295	58.0	6224
	Rent from LA/HA	21.5	1610	30.6	3283
	Rent privately	9.6	720	7.7	821
	Other (incl. with parents)	11.4	850	3.7	399
Country /Electoral Ward Deprivation	England – Advantaged	25.3	1897	24.6	2644
	England – Disadvantaged	24.9	1867	23.9	2571
	England – Ethnic	11.1	830	14.2	1531
	Wales – Advantaged	4.5	337	4.5	482
	Wales – Disadvantaged	10.9	813	10.1	1086
	Scotland – Advantaged	6.5	490	5.9	633
	Scotland –Disadvantaged	7.1	532	6.0	641
	N. Ireland – Advantaged	3.7	279	4.0	432
	N. Ireland Disadvantaged	5.9	445	6.8	731
Smoking Behaviour	Smoked During Pregnancy	15.8	1182	16.0	1713
	Did Not Smoke	84.2	6302	84.0	9030
Pregnancy Complications	No pregnancy comp	62.1	4651	62.7	6737
	Complications not associated with induction	17.7	1326	19.2	2066
	Complications associated with induction	15.5	1160	13.1	1415
	Other	4.7	353	5.0	533
Maternal BMI	Low (<18.5)	7.5	520	5.0	488
	Normal (18.5–24.9)	68.2	4737	63.1	6130
	High (≥25.0)	24.3	1690	31.9	3105
Infant Birth Weight	Low (<2500 g)	7.5	558	5.9	632
	Normal (2500-4000gs)	84.0	6287	81.7	8774
	High (>4000 g)	8.5	639	12.4	1333
Infant Gestational Age	259 days or less	10.5	788	10.6	1135
	260–272 days	14.0	1052	18.2	1960
	273–286 days	47.7	3575	50.1	5386
	287–293 days	23.4	1756	17.9	1926
	294 days or more	4.3	319	3.2	344

Fewer nulliparous women were married and more were cohabiting or single or divorced than their multiparous counterparts. In addition, a slightly higher percentage of nulliparous women had higher/first degrees (19.0%) than multiparous women (13.4%), with a lower percentage of nulliparous women reporting leaving education before their GCSEs (13.8%) than those women in the multiparous group (23.5%). A higher proportion of nulliparous women were in the highest income quintile and in managerial or professional occupations. Women in both groups were relatively equally represented across MCS country strata, with the proportions of respondents in each strata nearly identical.

Nulliparous and multiparous women had fairly similar percentage distributions across smoking behaviour, pregnancy and labour complications, infant birth weight, and gestational age in days. The groups differed slightly in maternal BMI, with fewer nulliparous women reporting pre-pregnancy BMIs of ≥25.0 (24.3%) than multiparous respondents (31.9%).

### Bivariate analysis

Pearson’s chi square tests were performed on the association between the explanatory variables and the likelihood of labour induction among the nulliparous and multiparous groups (Table 2).

**Table 2:** Bivariate Association between Explanatory Variables and Risk of Labour Induction among Nulliparous and Multiparous Women

		Nulliparous		Multiparous	
		%	*P* Value	%	*P* Value
Maternal Age	19 years and under	36.3	0.437	27.2	0.348
	20–25 years old	36.1		27.3	
	26–30 years old	35.4		27.7	
	31–35 years old	37.8		26.5	
	36 years and older	38.4		27.6	
Maternal Ethnicity	White	36.8	0.090	28.2	0.053
	Indian	34.2		25.8	
	Pakistani/Bangladeshi	35.4		23.3	
	Black/Black British	33.5		21.2	
	Other	31.0		20.7	
Maternal Marital Status	Legally Married	36.0	0.031	26.5	0.047
	Cohabiting	36.0		28.9	
	Single/Divorced	38.0		28.2	
Maternal Education	Higher/first degrees	34.0	0.337	20.8	0.000
	Diplomas in higher ed	36.0		28.1	
	A/O Levels (GSCE A-C)	37.5		28.1	
	Other (incl. GCSE D-G)	35.7		26.7	
	None	37.1		29.4	
Maternal Occupation	Managerial/professional	35.5	0.054	25.0	0.020
	Intermediate	35.7		26.8	
	Self-employed	38.6		26.3	
	Lower supervisor	42.6		25.5	
	Semi-routine/Routine	37.3		28.9	
	None	33.9		27.6	
Income Quintile	Lowest Quintile	36.4	0.534	28.9	0.000
	Second Quintile	38.1		28.0	
	Third Quintile	36.7		27.7	
	Fourth Quintile	35.8		25.0	
	Highest Quintile	35.3		23.9	
Housing Tenure	Own outright/mortgage	35.9	0.341	26.2	0.001
	Rent from LA/HA	36.4		29.1	
	Rent privately	36.6		27.0	
	Other (incl. with parents)	38.6		28.9	
Electoral Ward Deprivation	England – Advantaged	34.0	0.001	23.8	0.000
	England – Disadvantaged	35.8		26.4	
	England – Ethnic	34.3		22.7	
	Wales – Advantaged	33.5		24.9	
	Wales – Disadvantaged	35.6		28.8	
	Scotland – Advantaged	38.9		29.1	
	Scotland – Disadvantaged	41.1		32.0	
	Northern Ireland – Adv	50.5		37.7	
	Northern Ireland – Disadv	39.6		39.1	
Smoking Behaviour	Did Not Smoke	36.5	0.569	29.6	0.253
	Smoked During Pregnancy	36.4		26.8	
Pregnancy Complications	No preg complications	32.6	0.000	24.7	0.000
	Complications not associated with induction	36.5		30.1	
	Complications associated with induction	50.3		34.4	
	Other	40.5		29.1	
Maternal BMI	Low (<18.5)	33.7	0.000	26.1	0.000
	Normal (18.5–24.9)	34.1		26.1	
	High (≥25.0)	43.6		30.2	
Infant Birth Weight	Low (<2500 g)	32.1	0.000	29.6	0.000
	Normal (2500-4000 g)	35.4		25.8	
	High (>4000 g)	50.3		36.0	
Infant Gestational Age	259 days or less	30.4	0.000	25.2	0.000
	260–272 days	31.2		25.4	
	273–286 days	27.8		21.1	
	287–293 days	53.1		42.4	
	294 days or more	72.4		54.9	

Across all levels of all variables included in the bivariate analyses, a higher percentage of nulliparous women experienced labour induction than did multiparous women. Among nulliparous women, marital status (*p* < 0.05) and country/local area deprivation (*p* < 0.01) were found to have a significant association with induction, but educational qualifications, occupation before pregnancy, income quintile, and housing tenure did not. Conversely, each of the socioeconomic variables had significant relationships with labour induction for multiparous women.

While smoking in pregnancy did not have a significant association with the risk of induction in either group of women, each of the other maternal or infant health variables did have a significant relationship with induction in both groups. Pregnancy complications, maternal BMI, infant birth weight, and gestational age in days were strongly related to labour induction.

### Multivariate findings

The results of the logistic regression models for nulliparous and multiparous women are presented in Tables 3 and 4. While the addition of infant and maternal health variables in Model 2 changes the significance of the relationship between maternal educational qualifications and labour induction for nulliparous women, there is no difference in the direction or magnitude of associations presented in Models 1 and 2 for multiparous women.

**Table 3:** Odds Ratios for Logistic Regression of Labour Induction: Nulliparous Women

	Model 1 *N* = 6723			Model 2 *N* = 6305		
	Odds Ratio	95% CI	*p*-value	Odds Ratio	95% CI	*p*-value
Maternal Age						
19 years and under	0.721*	0.551, 0.943	0.017	0.755	0.561, 1.015	0.063
20–25 years old	0.795	0.630, 1.003	0.053	0.722*	0.560, 0.932	0.012
26–30 years old	0.840	0.677, 1.041	0.111	0.766*	0.606, 0.970	0.027
31–35 years old	0.961	0.768, 1.201	0.726	0.911	0.713, 1.163	0.454
36 years and older	Ref	Ref	Ref	Ref	Ref	Ref
Maternal Ethnicity						
White	Ref	Ref	Ref	Ref	Ref	Ref
Indian	0.910	0.624, 1.326	0.623	1.367	0.898, 2.081	0.144
Pakistani/Bangladeshi	0.971	0.697, 1.353	0.863	1.265	0.865, 1.850	0.225
Black/Black British	0.886	0.628, 1.251	0.493	0.876	0.589, 1.303	0.514
Other	0.833	0.597, 1.162	0.282	1.040	0.726, 1.490	0.832
Maternal Marital Status						
Legally Married	Ref	Ref	Ref	Ref	Ref	Ref
Cohabiting	1.027	0.901, 1.172	0.687	1.043	0.902, 1.206	0.567
Unpartnered	0.916	0.703, 1.194	0.516	0.952	0.713, 1.272	0.740
Maternal Education						
Higher/first degrees	Ref	Ref	Ref	Ref	Ref	Ref
Diplomas in higher education	1.104	0.907, 1.344	0.323	1.136	0.918, 1.408	0.241
A/O Levels (GSCE A-C)	1.145	0.985, 1.331	0.078	1.200*	1.107, 1.415	0.031
Other (incl. GCSE D-G)	1.082	0.883, 1.325	0.448	1.208	0.965, 1.511	0.098
None	1.202	0.960, 1.506	0.109	1.353*	1.054, 1.737	0.018
Electoral Ward Deprivation						
England – Advantaged	Ref	Ref	Ref	Ref	Ref	Ref
England – Disadvantaged	1.074	0.929, 1.241	0.336	1.059	0.904, 1.240	0.480
England – Ethnic	1.154	0.902, 1.477	0.256	1.200	0.910, 1.581	0.196
Wales – Advantaged	0.907	0.703, 1.170	0.452	0.850	0.642, 1.126	0.257
Wales – Disadvantaged	1.101	0.913, 1.328	0.312	1.098	0.896, 1.346	0.367
Scotland – Advantaged	1.203	0.975, 1.483	0.085	1.309*	1.041, 1.646	0.021
Scotland – Disadvantaged	1.303**	1.060, 1.603	0.012	1.393**	1.112, 1.745	0.004
N. Ireland – Advantaged	2.021***	1.563, 2.614	0.000	2.560***	1.947, 3.366	0.000
N. Ireland – Disadvantaged	1.200	0.953, 1.509	0.121	1.377*	1.072, 1.768	0.012
Pregnancy Complications						
No pregnancy complications				Ref	Ref	Ref
Complications not associated with induction				1.276**	1.101, 1.479	0.001
Complications associated with induction				2.653***	2.281, 3.085	0.000
Other				1.409**	1.097, 1.809	0.007
Maternal BMI						
Low (< 18.5)				Ref	Ref	Ref
Normal (18.5–24.9)				0.971	0.771, 1.221	0.799
High (≥25.0)				1.305*	1.109, 1.670	0.035
Infant Birth Weight						
Low (< 2500 g)				0.999	0.776, 1.286	0.995
Normal (2500–4000 g)				Ref	Ref	Ref
High (> 4000 g)				1.325**	1.094, 1.603	0.004
Infant Gestational Age						
259 days or less				0.954	0.764, 1.191	0.676
260–272 days				1.044	0.879, 1.240	0.624
273–286 days				Ref	Ref	Ref
287–293 days				3.058***	2.675, 3.500	0.000
294 days or more				8.049***	6.039, 10.727	0.000

Model 1 was adjusted for maternal occupation, housing tenure, and income quintile

Model 2 was adjusted for maternal occupation, housing tenure, income quintile, and smoking behaviour

*P < 0.05 **P < 0.01 ***P < 0.001

**Table 4:** Odds Ratios for Logistic Regression of Labour Induction: Multiparous Women

	Model 1 *N* = 9293			Model 2 *N* = 8616		
	Odds Ratio	95% CI	*p*-value	Odds Ratio	95% CI	*p*-value
Maternal Age						
19 years and under	0.863	0.582, 1.281	0.466	0.918	0.592, 1.422	0.701
20–25 years old	0.937	0.788, 1.115	0.466	0.955	0.791, 1.153	0.631
26–30 years old	0.944	0.816, 1.092	0.440	0.957	0.818, 1.120	0.587
31–35 years old	0.921	0.801, 1.060	0.253	0.925	0.796, 1.075	0.308
36 years and older	Ref	Ref	Ref	Ref	Ref	Ref
Maternal Ethnicity						
White	Ref	Ref	Ref	Ref	Ref	Ref
Indian	1.068	0.758, 1.505	0.708	1.302	0.897, 1.890	0.164
Pakistani/Bangladeshi	0.728*	0.531, 0.997	0.048	0.719	0.504, 1.027	0.069
Black/Black British	0.785	0.578, 1.066	0.121	0.844	0.597, 1.191	0.334
Other	0.800	0.553, 1.157	0.236	0.881	0.595, 1.304	0.526
Maternal Marital Status						
Legally Married	Ref	Ref	Ref	Ref	Ref	Ref
Cohabiting	0.996	0.878, 1.130	0.954	1.025	0.894, 1.175	0.722
Unpartnered	1.225*	1.040, 1.443	0.015	1.246*	1.044, 1.486	0.015
Maternal Education						
Higher/first degrees	Ref	Ref	Ref	Ref	Ref	Ref
Diplomas in higher education	1.525***	1.239, 1.879	0.000	1.522***	1.220, 1.899	0.000
A/O Levels (GSCE A-C)	1.477***	1.255, 1.737	0.000	1.556***	1.308, 1.850	0.000
Other (incl. GCSE D-G)	1.397**	1.133, 1.724	0.002	1.477**	1.179, 1.850	0.001
None	1.540***	1.255, 1.890	0.000	1.791***	1.436, 2.233	0.000
Electoral Ward Deprivation						
England – Advantaged	Ref	Ref	Ref	Ref	Ref	Ref
England – Disadvantaged	1.045	0.909, 1.201	0.533	1.057	0.911, 1.227	0.466
England – Ethnic	0.952	0.750, 1.208	0.684	0.932	0.717, 1.212	0.601
Wales – Advantaged	1.050	0.832, 1.325	0.682	1.011	0.790, 1.293	0.933
Wales – Disadvantaged	1.177	0.990, 1.400	0.065	1.180	0.980, 1.420	0.081
Scotland – Advantaged	1.326**	1.087, 1.617	0.005	1.344*	1.086, 1.663	0.007
Scotland – Disadvantaged	1.411**	1.156, 1.723	0.001	1.434**	1.159, 1.774	0.001
N. Ireland – Advantaged	1.952***	1.566, 2.434	0.000	2.232***	1.769, 2.816	0.000
N. Ireland – Disadvantaged	1.989***	1.649, 2.399	0.000	2.322***	1.904, 2.833	0.000
Pregnancy Complications						
No pregnancy complications				Ref	Ref	Ref
Complications not associated with induction				1.424***	1.254, 1.617	0.000
Complications associated with induction				1.902***	1.648, 2.194	0.000
Other				1.343*	1.070, 1.686	0.011
Maternal BMI						
Low (< 18.5)				Ref	Ref	Ref
Normal (18.5–24.9)				0.909	0.709, 1.166	0.452
High (≥25.0)				1.075	0.832, 1.390	0.578
Infant Birth Weight						
Low (<2500 g)				1.340*	1.049, 1.711	0.167
Normal (2500–4000 g)				Ref	Ref	Ref
High (>4000 g)				1.300***	1.125, 1.502	0.000
Infant Gestational Age						
259 days or less				1.087	0.889, 1.329	0.415
260–272 days				1.241**	1.081, 1.424	0.002
273–286 days				Ref	Ref	Ref
287–293 days				2.806***	2.468, 3.190	0.000
294 days or more				5.443***	4.213, 7.032	0.000

Model 1 was adjusted for maternal occupation, housing tenure, and income quintile

Model 2 was adjusted for maternal occupation, housing tenure, income quintile, and smoking behaviour

*P < 0.05 **P < 0.01 ***P < 0.001

Results from fully adjusted Model 2 for both nulliparous and multiparous women highlight relationships between labour induction and maternal and infant demographics in the two groups. Nulliparous women who were aged 20–25 years old (OR: 0.722, CI: 0.560, 0.932, *p* = 0.012) or aged 26–30 years old (OR: 0.766, CI: 0.606, 0.970, *p* = 0.027) were less likely to experience labour induction than women 36 years of age and older. However, maternal age does not have a significant relationship with induction of labour for multiparous women. Additionally, while marital status was not a predictor of labour induction for nulliparous women, unpartnered multiparous women were more likely than legally married women to undergo labour induction (OR: 1.246, CI: 1.044, 1.486, *p* = 0.015). Echoing the results of bivariate analyses, occupation, housing tenure, and income quintile had no association with induction of labour in any of the models run for both groups.

The two parity groups have similar patterns of association between educational qualifications and labour induction. For nulliparous women in Model 2, women with A/O Levels (OR: 1.200, CI: 1.107, 1.415, *p* = 0.031) or no educational qualifications (OR: 1.353, CI: 1.054, 1.737, *p* = 0.018) are at higher risk of induction than those with higher and first degrees. In multiparous women, maternal education is one of the most important predictors of labour induction. Multiparous women with higher and first degrees were less likely to experience labour inductions than women with any other educational qualification (Diplomas in higher education OR: 1.522, CI: 1.220, 1.899, *p* < 0.001; A/O Levels and GCSE A-C OR: 1.556, CI: 1.308, 1.850, *p* < 0.001; Other qualifications including overseas and GCSE D-G OR: 1.477, CI: 1.179, 1.850, *p* = 0.001; None OR: 1.791, CI: 1.436, 2.233, *p* < 0.001).

Electoral ward deprivation had a comparable association with labour induction in both groups of women. Nulliparous women living in advantaged and disadvantaged areas of Scotland and Northern Ireland had an increased risk of labour induction compared to women living in advantaged areas of England (Advantaged Scotland OR: 1.309, CI: 1.041, 1.646, *p* = 0.021; Disadvantaged Scotland OR: 1.393, CI: 1.112, 1.745, *p* = 0.004; Advantaged Northern Ireland OR: 2.560, CI: 1.947, 3.366, *p* < 0.001; Disadvantaged Northern Ireland OR: 1.377, CI: 1.072, 1.768, *p* = 0.012). A similar relationship was apparent for the multiparous group: living in both advantaged and disadvantaged areas of Scotland and Northern Ireland placed women at greater risk of labour induction than living in advantaged areas of England (Advantaged Scotland OR: 1.344, CI: 1.086, 1.663, *p* = 0.007; Disadvantaged Scotland OR: 1.434, CI: 1.159, 1.774, *p* = 0.001; Advantaged Northern Ireland OR: 2.232, CI: 1.769, 2.816, *p* < 0.001; Disadvantaged Northern Ireland OR: 2.322, CI: 1.904, 2.833, *p* < 0.001). Overall, living in Northern Ireland placed women at greater risk of labour induction than living in any other country in the UK.

Regardless of parity, women who experienced complications during pregnancy were more likely to undergo induction of labour than were women who had no pregnancy complications, and a late or post term gestational age put women at higher risk of labour induction than being at term.

## Discussion

This study of the maternal and infant predictors of labour induction in the United Kingdom described several maternal demographic, socioeconomic, and health associations with induction of labour. While maternal health variables had relationships with labour induction similar to those that have been found in other countries, this paper presents some unique socioeconomic associations.

Income quintile and maternal occupation were not significant predictors of labour induction for women in the MCS. This is at odds with some previously published studies on childbirth intervention and labour induction, which have found that income-based measures of socioeconomic status have significant associations with induction of labour [18]. However, much of the research into predictors of childbirth intervention has been conducted in the United States, where differences in health care payment and provision may make the results difficult or even impossible to generalize to the UK. In the United Kingdom, where universal health care is established, it follows that some socioeconomic variables are not as profound an influence on health care practices as they are in the United States.

Maternal education and local area deprivation, both proxies of socioeconomic status, did have significant relationships with labour induction for both groups women, with the influence of education on risk of labour induction most salient in multiparous women. Multiparous women with higher educational qualifications were less likely to be induced than those with lower educational qualifications. This difference in labour induction risk by education may be due in part to varying conceptualizations of labour and birth in women with different educational backgrounds. It is possible that women with fewer educational qualifications viewed labour induction as a more standard part of the childbirth experience than did women with more educational qualifications. Previous studies have found that differences in childbirth experiences by socioeconomic status were related to the different expectations and preferences held by women in each group [19].

Another explanation for the significance of educational attainment in multiparous women is that women who had given birth at least once before the birth of the cohort baby may have drawn on their previous childbirth experience in addition to their education, making highly educated multiparous women more inclined to vocalize their preferences in childbirth. Studies have shown that educational attainment can influence a women’s perceived control over her health care and her ability to navigate the health care system available to her, and higher education has been linked to lower risk of labour induction and higher confidence in medical decision making in previous research [20, 14]. Previous research posits that an increase in educational attainment can lead to an increase in self-efficacy, which is “the belief that one can successfully accomplish a task and one’s estimation that if the task is accomplished, it will lead to specific outcomes” [21], meaning that women who are more educated may be able to more confidently advocate for themselves both before and during their labours. Women with greater feelings of self-efficacy have been found to be more positive about pregnancy and birth, and to feel less pain and use fewer interventions (such as epidural pain management) during labour [21, 22]. As the number of women in higher education has risen since 2000, future research into how education and parity influence maternal choice in childbirth in more recent cohorts would help illuminate the relationship between maternal self-efficacy and labour induction.

The significance of local area deprivation may shed light on the importance of access to quality services, access to the transportation to these services, the quality/interest of providers, and the types of social support in place in a woman’s life to allow her to make decisions about her health throughout pregnancy and care during childbirth. Even in countries where health care is made universally available, women in disadvantaged places may have to contend with busier clinics, longer wait times, lower quality interactions with medical professionals, trouble securing transportation to clinics, and a lack of social support, all of which makes accessing available care more difficult [23, 24].

This may be particularly true in Northern Ireland. Northern Ireland consistently has the highest rates of labour induction and caesarean section in the UK and in the Republic of Ireland [25]. In addition, according to a study by Abel et al. (2016), which adjusted Indices of Multiple Deprivation from each UK country in an effort to allow for the comparison of deprivation between countries, 37% of the population of Northern Ireland lived in places falling in most deprived fifth of the United Kingdom, making it the most deprived country in the UK [26]. The greater deprivation and higher rates of childbirth intervention documented in Northern Ireland are reflected in the greater risk of induction for women living in both disadvantaged and advantaged electoral wards in Northern Ireland found in this analysis. It may be that in Northern Ireland, women living in advantaged electoral wards are still disadvantaged when compared to women living in advantaged electoral wards in England, and that this relative disadvantage is evidenced by their greater risk of labour induction.

### Limitations

The present analyses were strengthened by the inclusion of many maternal demographic, socioeconomic, and health variables, and by the large, UK-wide sample offered in the Millennium Cohort Study. This broad sample, taken from each of the four UK countries, allowed for the analysis of induction risk factors for each country and for a comparison of the results to be made between countries. The division of the sample by parity helped to highlight differences between women who were experiencing their first births and women who had had other children, and potential reasons for these marked differences.

Perhaps the most critical data limitation was the age of the information in the MCS, as the data were collected in 2000–2001. The MCS was the best dataset available for the research undertaken here, in that it included the maternal demographic, socioeconomic, and health variables of interest and allowed for the generalization of results to each of the four countries of the United Kingdom. The age of the data may encourage questions about its relevance, but given that the core structure of NHS maternal health provision and NICE labour induction guidelines have remained very similar since 2001 [27, 28], and that there are no other comparable datasets in the United Kingdom, the MCS is the best option for conducting research into the risk of childbirth intervention across the whole of the United Kingdom.

A limitation in this study is that variables that could have bolstered the strength of the analyses are not available in the MCS dataset. The MCS contains no information about why a labour was induced, how the labour was induced (either intravenously or manually), or whether the labour “induction” was perhaps in fact a labour “augmentation,” with induction techniques utilized to speed up a slow labour. Previous literature examining childbirth interventions by socioeconomic status in countries with universal health care reports that for many interventions, risk is higher for women with lower socioeconomic status [9, 14, 15]. Therefore, while it is possible that the number of straightforward labour inductions were over reported in this sample, this is unlikely to have changed the direction of the relationships between socioeconomic status and labour intervention reported here. However, to the best of our knowledge, no previous research has examined the relationship between maternal socioeconomic status and labour augmentation specifically, meaning it is an avenue for further research. Future projects would benefit from a more nuanced definition of induction of labour. More detailed information about the labour inductions experienced by women in this sample would also help underline the associations between induction and various maternal indicators. These analyses also did not include variables concerning the duration of labour, which the literature reports could be linked to the risk of labour induction, or whether a woman had previously given birth by caesarean section. Previous operative birth could influence a multiparous women’s risk of induction, as past caesarean sections can complicate future labour inductions. Further research could benefit from addition of these maternal health variables into the models.

Additionally, given the significance of the association between induction of labour and the relative advantage or disadvantage of the location in which a woman lived, further analyses into the link between labour induction and socioeconomic status are indicated. Future analyses would be best served by examining labour induction in the context of the characteristics of health care providers, such hospitals or trusts, which could both allow more thorough quantitative spatial analyses to be performed, and provide much needed qualitative data about the experiences of individual women in varying health care contexts. A thorough examination of the mediators inherent to health care providers would allow future research to more fully understand what about a woman’s location made her more or less likely to undergo labour induction in the present analyses.

## Conclusion

The results presented above indicate that the risk of labour induction does indeed differ by socioeconomic status for women in the United Kingdom. Although nulliparous women are more likely to be induced, indicators of socioeconomic status such as maternal educational qualifications and electoral ward deprivation had more significant relationships with induction in multiparous women. The results of the present research highlight the importance of studying the influence of a woman’s environment and education on how she engages with health care practitioners and how she participates in medical decision-making.

## Data Availability

The datasets generated and/or analysed during the current study are available in the UK Data Service repository, https://beta.ukdataservice.ac.uk/datacatalogue/studies/study?id=4683. Some of the data that support the findings of this study are available from the UK Data Service but restrictions apply to the availability of these data, which were used under license for the current study, and so are not publicly available. Data are however available from the authors upon reasonable request and with permission of the UK Data Service.

## Abbreviations

BMI: Body mass index
MCS: Millenium Cohort Study
NISRA: Northern Ireland Statistics and Research Agency
ONS: Office of National Statistics
UK: United Kingdom
